# Traumatic left ventricular apical pseudoaneurysm in a young woman: a rare and life-threatening condition

**DOI:** 10.1093/ehjcr/ytag401

**Published:** 2026-06-02

**Authors:** Ouafae Zouhri, Yasmine Hajji, Amal Tazi Mezalek, Younes Cheikhaoui

**Affiliations:** Department of Cardiology, Cheikh Zaid International University Hospital, Allal El Fassi avenue, 10000 Rabat, Morocco; Department of Cardiology, Cheikh Zaid International University Hospital, Allal El Fassi avenue, 10000 Rabat, Morocco; Department of Cardiology, Cheikh Zaid International University Hospital, Allal El Fassi avenue, 10000 Rabat, Morocco; Department of Pediatric Cardiology, Cheikh Zaid International University Hospital, Allal El Fassi, 10000 Rabat, Morocco

Left ventricular (LV) pseudoaneurysm is a rare but life-threatening condition resulting from a contained rupture of the myocardial wall, in which blood is confined by the pericardium rather than by the myocardial tissue. Although most pseudoaneurysms are ischemic and occur after myocardial infarction,^1^ nonischemic etiologies—particularly traumatic ones—are exceptional.^2^

We report the case of a 24-year-old woman with no cardiovascular risk factors who presented with progressive dyspnea and right heart failure. Medical history was notable for a childhood fall with thoracic impact and a direct chest trauma (punch) that occurred five years prior to presentation that had not been medically evaluated. The electrocardiogram showed diffuse low-voltage QRS complexes, sinus tachycardia, a normal cardiac axis, and diffuse T-wave inversions.

Cardiac magnetic resonance imaging (MRI) identified an 8 mm apical wall rupture communicating with a large fluid-filled cavity measuring 200 × 100 mm, partially thrombosed (thrombus measuring, 65 × 45 mm), consistent with a giant LV pseudoaneurysm. Imaging revealed a giant apical LV pseudoaneurysm compressing the right chambers (*Figure 1*). Key features distinguishing this pseudoaneurysm from a true aneurysm included a narrow neck (neck-to-cavity ratio, 0.04, well below 0.5), discontinuity of the apical myocardium, and a cavity bounded solely by the pericardial sac without a myocardial wall. The hemodynamic impact is characterized by two mechanisms. First, a systolic flow diversion through the 8 mm neck into the pseudoaneurysmal sac, reducing effective stroke volume. Second, the giant dimensions of the sac exert a significant mass effect, causing mechanical compression of the right ventricle as visualized on both transthoracic echocardiography and cine-MRI. This compression restricts right heart filling, mimicking a localized restrictive or constrictive physiology

**Figure 1:**
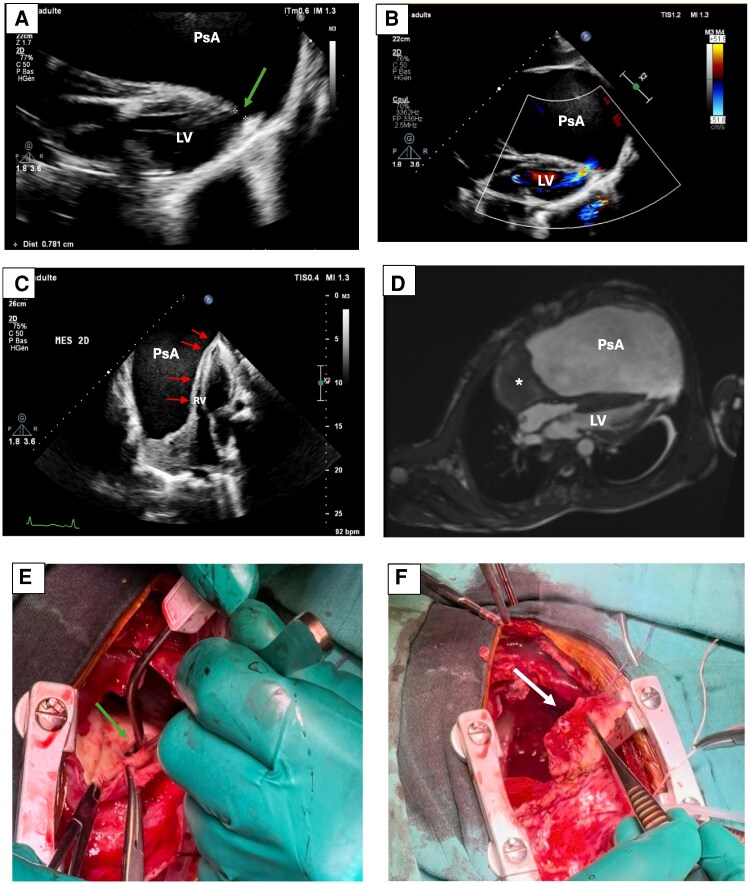
Multimodal assessment of a giant left ventricular pseudoaneurysm. (*A*) Transthoracic echocardiography (subcostal view) showing the left ventricular pseudoaneurysm, with the neck measuring 8 mm indicated by the green arrow. (*B*) Transthoracic echocardiography, Color Doppler demonstrating a systolic–diastolic flow across the neck. (*C*) Transthoracic echocardiography, four-chamber view, showing right heart chamber collapse caused by a left ventricular apical pseudoaneurysm. The red arrows indicate the significant mechanical compression of the right ventricle free wall, highlighting the mass effect exerted by the giant sac. (*D*) Cardiac magnetic resonance imaging (cine T1 sequence, four-chamber view) showing a partially thrombosed (asterisk) apical left ventricular pseudoaneurysm. (*E*) Intraoperative view showing the neck connecting the pseudoaneurysm (green arrow). (*F*) Intraoperative view showing the thickened pericardium indicated by the white arrow (pericardectomy). (Moving image available in the online version of the article.)

Surgical exploration showed a thickened, calcified pericardium consistent with chronic pericarditis. The pseudoaneurysm cavity, formed by organized pericardium, was repaired with a bovine pericardial patch reinforced by Dacron strips, followed by pericardiectomy. Postoperative recovery was uneventful, and echocardiography confirmed complete resolution.

This case illustrates an exceptional traumatic LV pseudoaneurysm in a young woman and highlights the paradoxical protective role of chronic pericarditis in preventing fatal rupture. Although the chronological relationship between the chronic pericarditis and the pseudoaneurysm remains uncertain, the marked sac expansion and absence of constrictive features on MRI suggest that the pericardial thickening was more likely a secondary inflammatory reaction than a preexisting protective condition. This case underscores the importance of early diagnosis using multimodal imaging and timely surgical management to ensure favorable outcomes.

**Patient consent:** Written informed consent for publication of the clinical information and images were obtained from the patient in accordance with COPE guidelines.

## Supplementary Material

ytag401_Supplementary_Data

## Data Availability

This case is based on the image presented in the article as well as additional images and clinical information. A portion of these data can be provided in deidentified form upon reasonable request. However, some raw data cannot be shared due to confidentiality restrictions.
