# Supplementary figures and images for: Traumatic left ventricular apical pseudoaneurysm in a young woman: a rare and life-threatening condition
Source: Eur Heart J Case Rep. 2026 Jun 2;10(6):ytag401. doi: 10.1093/ehjcr/ytag401 (PMC13249112; doi:10.1093/ehjcr/ytag401)

[
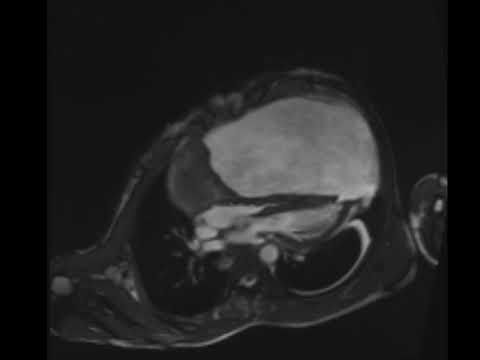
](https://www.youtube.com/embed/ye8LoSgtWew?feature=oembed)

Supplement: ytag401_Supplementary_Data [file ytag401_supplementary_data.docx]
